# Sliding and pressure evaluation on conventional and V-shaped seats of reclining wheelchairs for stroke patients with flaccid hemiplegia: a crossover trial

**DOI:** 10.1186/1743-0003-8-40

**Published:** 2011-07-16

**Authors:** Hsiu-Chen Huang, Cheng-Hsin Yeh, Chi-Myn Chen, Yu-Sheng Lin, Kao-Chi Chung

**Affiliations:** 1 Institute of Biomedical Engineering, National Cheng Kung University, Tainan, Taiwan; 2 Department of Physical Medicine and Rehabilitation, Chia-Yi Christian Hospital, Chia-Yi, Taiwan

## Abstract

**Background:**

Reclining wheelchairs are commonly used to transport elderly stroke patients in Taiwan. However, there is concern that the patient's body in the wheelchair often slides forward when they return to a seated position, increasing the sitting pressure. Therefore, a novel reclining wheelchair with an ergonomic "V-Seat" was designed to prevent forward sliding and pressure sores. The use of these reclining chairs by stroke patients has not yet been studied. Thus, we investigated the effects of V-shaped and conventional seats in reclining wheelchairs on the extent of forward sliding and on the sitting pressure of stroke patients with flaccid hemiplegia and of able-bodied elders.

**Methods:**

We recruited 13 able-bodied elders and 11 stroke patients with flaccid hemiplegia and performed 5 reclining cycles in both types of wheelchair. The amount of sliding along the backrest (BS) plane and the seat (SS) plane, the mean sitting pressure (MP), and the sacral peak pressure (SPP) of the subjects were recorded. We used the Wilcoxon signed-rank test to compare the BS, SS, MP, and SPP in wheelchairs with conventional and V-shaped seats, and we used the Wilcoxon rank sum test to compare the differences in BS and SS between stroke patients and able-bodied elders in both types of reclining wheelchair.

**Results:**

The BS, SS, and SPP of stroke patients were significantly lower in the wheelchairs with V-shaped seats than in conventional wheelchairs in most comparisons; however, the BS of able-bodied elders was higher in V-shaped seats than in conventional seats. The SS and SPP of stroke patients were significantly higher than those of able-bodied elders in both types of reclining wheelchair, and the BS of stroke patients was significantly higher than that of able-bodied elders only in conventional reclining wheelchairs.

**Conclusions:**

The use of V-shaped seats in reclining wheelchairs can help reduce the forward sliding and sacral peak pressure of stroke patients with flaccid hemiplegia. The back displacement of able-bodied subjects when using both conventional and V-shape seats in reclining positions differs from the back displacement of stroke patients with flaccid hemiplegia when using such seats. These results are of paramount value and should be considered when prescribing the use of reclining wheelchairs to subjects with flaccid hemiplegia.

## Background

Reclining wheelchairs are commonly used to transport stroke patients in Taiwan. For example, from 2007 to 2009, reclining wheelchairs accounted for 66.4% of specialized wheelchair prescriptions at our assistive devices/technology center, and 58.5% of the reclining wheelchair users were stroke patients [1]. Most reclining wheelchairs are light-weight, inexpensive, foldable, and commercially available. Moreover, reclining wheelchairs provide better trunk support [2] to stroke patients who suffer from hemiplegia and poor sitting balance and/or tolerance and also help reduce the sitting pressure [3] of elderly stroke patients, who are susceptible to pressure sores [4].

**Figure 1:**
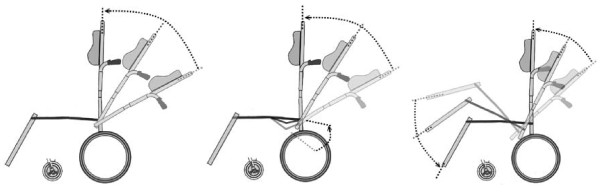
**The comparison between conventional reclining wheelchairs, reclining wheelchairs with V-shaped seats, and tilt-in-space wheelchairs (Left to right)**. The rear halves of the V-shaped seat and the tilt-in-space seat are similar, but the V-shaped seat differs from the traditional tilt-in-space seat in the following ways: 1) The front half of the seat is fixed, 2) The degree of downward inclination (maximum of 20 degrees) is less than that of the tilt-in-space seat, and 3) The seat-to-back angle varies.

However, there is concern regarding the use of reclining wheelchairs by stroke patients. The patient's body often slides forward in the wheelchair when returning to a seated position from a reclined position. This sliding leads to a sacral sitting posture and results in increased sacral shear stress, predisposing the patient to a sacral pressure sore [5,6] and requiring caregivers to frequently reposition their patients. Therefore, with the goal of preventing forward sliding and pressure sores, a novel ergonomic "V-Seat" has been designed. This seat "sinks" at the backrest at a downward angle of up to 20 degrees, whereas the backrest itself reclines up to 160 degrees (Figure 1). However, the use of these chairs by stroke patients has not yet been studied.

We investigated the effects of V-shaped and conventional seats in reclining wheelchairs on the forward sliding and sitting pressure of stroke patients with flaccid hemiplegia and able-bodied elders. We hypothesized that 1) able-bodied elders and flaccid hemiplegic patients have different mechanisms of forward sliding and interface pressure when using the V-shaped seats, and 2) the "V-Seat" reclining wheelchair can reduce the degrees of forward sliding and sitting pressure of stroke patients with flaccid hemiplegia.

## Methods

### Subject description

We recruited able-bodied elders and non-ambulatory stroke patients with flaccid hemiplegia to investigate the difference between wheelchairs with conventional and V-shaped seats. To minimize anthropometric differences among the subjects, the inclusion criteria were a weight of 40-70 kg, a body length of 140-170 cm, and an age of 60-85 years. Stroke patients who had consciousness disturbances, severe cognitive deficits (e.g., becoming agitated or disobedient), aphasia, or bilateral hemiplegia were excluded from this study. The demographic data of the study subjects were recorded.

### Equipment and materials

**Figure 2:**
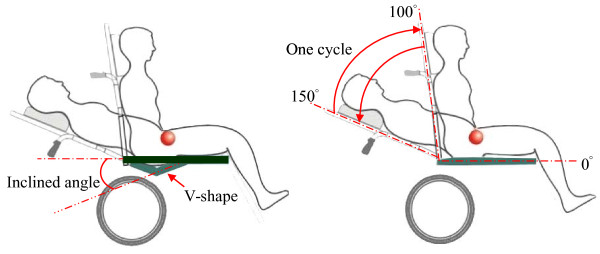
**The difference in seat mechanics between wheelchairs with conventional (Right) and V-shaped seats (Left)**. Cyclic tests were performed by reclining the seat back angle (SBA) from 100 to 150 degrees and then back to 100 degrees.

We used wheelchairs with conventional and V-shaped seats in this study (Figure 2). The wheelchair with a V-shaped seat (16" Model, KARMA MEDICAL PRODUCTS CO., LTD.) features an anti-sliding V-shaped seat that "sinks" to a maximum downward angle of 20 degrees, whereas the backrest can recline up to 160 degrees. The conventional wheelchair was also 16" wide (KARMA MEDICAL PRODUCTS CO., LTD.). Both types of wheelchairs were equipped with the same backrest, legrest and foam cushions to ensure that the seat mechanics were the only difference between the wheelchairs. To specifically measure the effect of V-shaped seats versus conventional seats, the armrests were removed for this study. A sensor system (X3 PX100:36.36.02, XSENSOR) was used to collect pressure data at the seat interfaces. The sensor system was a flexible pad with a 46 cm × 46 cm sensing area containing 1296 (36 × 36) sensing points. The measured pressure range was 10-200 mmHg. The accuracy of the pressure measurement was ± 10% of the full scale.

### Experimental protocol

**Figure 3:**
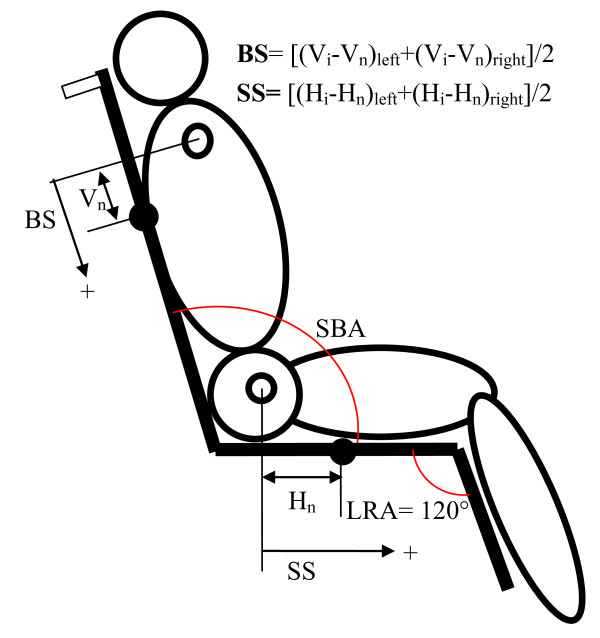
**The definition of sliding along the backrest (BS) and sliding along the seat (SS)**. Vn and Hn were recorded when the seat back angle (SBA) was at 100 degrees. The hollow circle and solid circle represent the anatomical markers and wheelchair markers, respectively.

At the beginning of testing, the geometries of both types of wheelchair were adjusted to a seat back angle (SBA) of 100 degrees and a legrest angle (LRA) of 120 degrees (Figure 3). The subjects randomly selected one of the wheelchairs without knowing its type, as the wheelchairs look almost identical. Each subject was asked to sit comfortably on the wheelchair, and their starting position was marked to allow measurements of the sagittal motion of his or her body. Four anatomical markers (bilateral acromion and greater trochanter) and four wheelchair markers on both sides of the backrest and seat were used. Five reclining cycles were performed by reclining the SBA from 100 to 150 degrees and then back to 100 degrees at a constant rate (Figure 2). If the subjects could not continue sitting, due to intolerance or to the danger of sliding out of the wheelchair, the experiment was stopped for safety reasons. Between each cycle, the subjects were asked to relax for a one-minute break. The geometric and mechanical data were measured by a well-trained physical therapist.

### Geometric and mechanical parameters

The geometric parameters include the degrees of sliding along the backrest (BS) and sliding along the seat (SS), which are standard measures also used by Aissaoui et al. [7] and are defined as follows (Figure 3):

where V_i _and V_n _are the distances perpendicular to the backrest support plane between the backrest marker and the acromion marker in its initial position (i) and after a number of cycles (n). H_i _and H_n _are the distances perpendicular to the seat support plane between the seat marker and the trochanter marker in its initial position (i) and after a number of cycles (n). The sliding along the backrest (BS) and along the seat (SS) were calculated by averaging the values measured on the left and right sides to represent the midline sagittal sliding. In addition, mechanical parameters were recorded to evaluate the pressure distribution at the seat interface. These parameters include the mean pressure (MP) in the total contact area (> 5.2 mmHg) and the sacral peak pressure (SPP).

### Statistical methods

The results are presented as the means ± standard deviations, and comparisons of the BS, SS, MP, and SPP measured in wheelchairs with conventional and V-shaped seats were made using the Wilcoxon signed-rank test. The Wilcoxon rank sum test was used to compare the difference in BS and SS between stroke patients with flaccid hemiplegia and able-bodied elders in both types of reclining wheelchairs. This difference indicates the effect of hypotonicity on postural control. All statistical tests were conducted using the Statistical Package for the Social Sciences (SPSS) 15.0 for Windows with a two-tailed significance level of 0.05.

### Ethical issues

The study's protocol was approved by the institutional review board of the Chia-Yi Christian Hospital. All subjects provided informed consent.

## Results

### Demographics

Twenty-five elders participated in this study. The data from one subject is not included due to an extremely unstable sitting condition. Of the 24 participating subjects, 10 were men and 14 were women. Of the 11 patients with flaccid hemiplegia, 7 suffered from left-sided hemiplegia and 4 from right-sided hemiplegia. No significant differences between the able-bodied and flaccid hemiplegic groups were found in age, weight, or height (Table 1).

**Table 1:** Demographics of able-bodied and flaccid hemiplegic subject groups

Group		Able-bodied	Flaccid	P
Gender	Male	5	5	> 0.95
	Female	8	6	
				
Age	Mean	71.9	72.6	0.865
(years)	SD	5.8	8.4	
				
Weight	Mean	62.7	58.4	0.207
(Kg)	SD	6.6	10.7	
				
Height	Mean	160.0	157.5	0.459
(cm)	SD	7.2	8.8	

### Geometric outcomes (BS and SS)

Table 2 shows the variations in BS between the subject groups and between the wheelchair types. In general, the average BS values of these groups were positive, indicating that the median location of the bilateral acromion is displaced downward during the reclining cycles. In the conventional wheelchairs, the BS of the flaccid hemiplegic group was significantly higher than that of the able-bodied group over the first 3 cycles, whereas no significant differences were found between the two groups in wheelchairs with V-shaped seats. For the flaccid hemiplegic subjects, wheelchairs with V-shaped seats caused less BS than the conventional wheelchairs at a statistically significant level after the third cycle, with only a marginal difference after the first cycle. For able-bodied subjects, the BS in wheelchairs with V-shaped seats was significantly higher than in conventional reclining wheelchairs after the fifth cycle.

**Table 2:** Measurement of sliding along the backrest plane by subject groups and wheelchair (WC) types

Cycles	V-seat WC							Conventional WC							Flaccid		Able-bodied	
	
	Flaccid			Able-bodied			p	Flaccid			Able-bodied			p	**V-seat vs. Conv**.	p	**V-seat vs. Conv**.	p
								
	n	Mean	SD	n	Mean	SD		n	Mean	SD	n	Mean	SD		**Diff**.		**Diff**.	
1	11	8.3	12.2	13	11.3	8.7	0.691	11	20.6	15.2	13	4.0	8.2	0.005*	-12.3	0.068	7.4	0.087
2	11	16.9	13.4	13	12.9	11.4	0.494	11	32.6	22.5	13	5.0	11.3	0.001*	-15.6	0.110	7.9	0.108
3	9	15.8	15.0	13	12.7	11.7	0.357	8	41.6	27.0	13	7.5	14.0	0.002*	-25.8	0.036*	5.2	0.248
4	5	12.2	5.6	13	13.3	13.6	0.924	4	33.6	23.5	13	9.0	11.1	0.130†	-21.4	0.465	4.3	0.263
5	4	14.6	9.3	13	13.9	12.9	0.956	4	33.9	24.3	13	6.5	11.4	0.130†	-19.3	> 0.95	7.5	0.043*

*p < 0.05 †The nonparametric tests lack statistical power with small sample size.

Table 3 shows the variations in sliding along the seat plane (SS) between the subject groups and the wheelchair types. Positive values of SS indicate forward displacements of the median of the greater trochanter along the seat plane measured on both sides, whereas negative values indicate rearward displacements from the initial position. In both types of wheelchairs, flaccid hemiplegic subjects slid significantly further forward than able-bodied subjects (except after cycle 4, which may be due to the small sample size of the flaccid hemiplegic group). Flaccid hemiplegic subjects slid significantly less further forward in V-shaped seat wheelchairs than in conventional wheelchairs over the first 3 cycles, whereas for able-bodied subjects, there were significant differences between the two types of wheelchairs except after the first cycle, and there was only a marginal difference after cycle 4.

**Table 3:** Measurement of sliding along the seat plane by subject groups and wheelchair (WC) types

Cycles	V-seat WC							Conventional WC							Flaccid		Able-bodied	
	
	Flaccid			Able-bodied			p	Flaccid			Able-bodied			p	**V-seat vs. Conv**.	p	**V-seat vs. Conv**.	p
								
	n	Mean	SD	n	Mean	SD		n	Mean	SD	n	Mean	SD		**Diff**.		**Diff**.	
1	10	6.6	8.7	13	-1.7	5.4	0.011*	10	25.4	19.5	13	2.4	13.7	0.002*	-18.8	0.007*	-4.1	0.286
2	10	9.2	9.7	13	-3.7	7.5	0.002*	10	45.9	28.8	13	4.7	12.7	< 0.001*	-36.7	0.003*	-8.4	0.012*
3	8	12.1	12.5	13	-3	7.8	0.004*	8	54.8	30.6	13	4.4	14.7	< 0.001*	-42.7	0.012*	-7.4	0.045*
4	5	7.7	8.9	13	-2.1	8.5	0.117	4	44.6	13.4	13	3.9	13.2	0.002*	-36.9	0.068†	-6	0.055
5	4	13.8	9.9	13	-2.2	8.6	0.023*	4	54.6	16.7	13	6.4	16	0.002*	-40.8	0.109†	-8.6	0.006*

*p < 0.05 †The nonparametric tests lack statistical power with small sample size.

**Figure 4:**
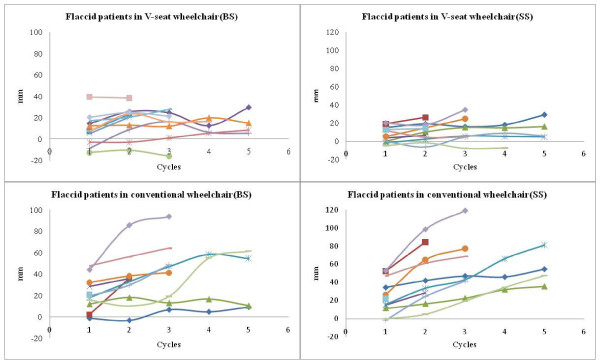
**The distribution of sliding along the backrest and seat planes by flaccid hemiplegic subjects**. BS and SS represent the sliding along the backrest (BS) plane and the sliding along the seat (SS) plane, respectively.

Figure 4 shows the distributions of BS and SS of flaccid hemiplegic subjects in both wheelchair types. Some data points could not be collected after the third cycle, as some of the flaccid hemiplegic subjects could not participate for the entire experimental procedure. This missing data results in a small sample size and reduces the statistical power. The ranges of BS were between -16 and 40 mm in the wheelchairs with V-shaped seats and between -2 and 94 mm in the conventional wheelchairs. The ranges of SS were between -8 and 35 mm in the wheelchairs with V-shape seats and between 0 and 120 mm in the conventional wheelchairs. Overall, the ranges of sliding along both the backrest and seat planes were larger in the conventional wheelchairs than in the wheelchairs with V-shaped seats.

### Mechanical outcome (MP, SPP)

The comparisons of the mean seat pressures of the subject groups and wheelchair types are shown in Table 4. Overall, no significant differences were found among the groups. The mean pressure slightly increased with the number of completed reclining cycles and varied from 35.8 to 40.0 mmHg.

**Table 4:** Mean pressure (MP, mmHg) measurements by subject groups and wheelchair (WC) types

Cycles	V-seat WC							Conventional WC							Flaccid		Able-bodied	
	
	Flaccid			Able-bodied			p	Flaccid			Able-bodied			p	**V-seat vs. Conv**.	p	**V-seat vs. Conv**.	p
								
	n	Mean	SD	n	Mean	SD		n	Mean	SD	n	Mean	SD		**Diff**.		**Diff**.	
0	11	36.1	3.9	13	36.7	1.9	0.277	11	35.8	3.2	13	36.3	2.3	0.776	0.3	0.859	0.4	0.311
1	11	37.2	3.7	13	38.3	1.8	0.186	11	37.1	2.9	13	38.0	2.0	0.277	0.1	0.959	0.3	0.507
2	11	37.3	3.7	13	38.9	1.9	0.055	11	38.2	3.0	13	38.6	2.0	0.910	-0.8	0.182	0.3	0.552
3	9	38.2	3.5	13	39.4	1.8	0.051	8	38.8	3.2	13	39.1	1.9	0.916	-0.7	0.401	0.3	0.600
4	5	37.5	1.6	13	39.7	2.1	0.075	4	37.0	2.2	13	39.3	2.0	0.130	0.5	0.715	0.4	0.507
5	4	38.0	2.1	13	39.9	2.0	0.163	4	37.2	2.0	13	39.7	2.0	0.130	0.7	1.000	0.2	0.754

Table 5 shows that subjects with flaccid hemiplegia were susceptible to higher sacral peak pressures than able-bodied subjects in both types of wheelchair, and significant differences were found after every cycle (marginal difference after cycle 4 in conventional wheelchairs). For subjects with flaccid hemiplegia, the sacral peak pressures measured in the initial measurement and after the first 3 cycles were significantly lower in the wheelchairs with V-shaped seats than in conventional wheelchairs (marginal significance after cycle one). For able-bodied subjects, the SPP was less in the wheelchairs with V-shaped seats than in the conventional wheelchairs; this difference was apparent at the initial measurement and after cycle 4, and there was a marginal difference after cycle one.

**Table 5:** Sacral peak pressure (SPP, mmHg) measurements by subject groups and wheelchair (WC) types

	V-seat WC							Conventional WC							Flaccid		Able-bodied	
	
Cycles	Flaccid			Able-bodied			p	Flaccid			Able-bodied			p	**V-seat vs. Conv**.	p	**V-seat vs. Conv**.	p
								
	n	Mean	SD	n	Mean	SD		n	Mean	SD	n	Mean	SD		**Diff**.		**Diff**.	
0	11	62.2	33.3	13	41.0	4.6	0.03*	11	75.4	34.8	13	51.1	10.7	0.047*	-13.2	0.041*	-10.2	0.013*
1	11	72.0	37.7	13	44.3	6.6	0.018*	11	89.4	48.5	13	50.2	9.0	0.011*	-17.4	0.062	-5.9	0.064
2	11	72.9	40.1	13	45.6	8.1	0.026*	11	99.7	47.3	13	50.6	9.8	0.001*	-26.8	0.005*	-5.0	0.133
3	9	78.4	39.6	13	46.5	10.2	0.009*	8	116.3	49.1	13	51.6	13.1	< 0.001*	-38.0	0.012*	-5.1	0.249
4	5	69.7	23.6	13	45.7	9.5	0.014*	3†	78.4	23.5	13	51.6	10.1	0.057	-8.8	0.285	-5.9	0.039*
5	4	81.7	32.8	13	45.3	9.4	0.023*	4	102.4	44.2	13	51.2	9.4	0.006*	-20.6	0.180	-5.9	0.064

*p < 0.05 †There is one missing data at cycle 4.

## Discussion

Our results indicate that stroke patients with flaccid hemiplegia are more vulnerable to forward sliding along the seat plane and are, therefore, subject to higher sacral peak pressures (SPP) than able-bodied elders in both types of wheelchairs. V-shaped seats noticeably reduce the forward sliding of stroke patients and also tend to decrease forward sliding in able-bodied elders, indicating that V-shaped seats can help prevent wheelchair users from sacral sitting. Moreover, these seats can also help prevent the development of sacral pressure sores, as evidenced by the significantly lower SPP measured in the wheelchairs with V-shaped seats compared to the conventional type.

Proper seating/positioning of non-ambulatory stroke patients with flaccid hemiplegia who need to remain seated for long periods of time is difficult to achieve because the patients often have poor trunk control and poor sitting tolerance. Reclining wheelchairs are commonly used for non-ambulatory stroke patients with flaccid hemiplegia in hospital settings and nursing facilities because they serve not only as transportation tools but also as assistive devices for better positioning in long-term care. Our study provides important data regarding forward sliding and sitting pressure in reclining wheelchairs for stroke patients, and these data may provide valuable information for selecting the appropriate reclining wheelchair.

Previous research on wheelchair design recruited able-bodied subjects to collect data about seating position, but these results cannot be directly applied to patients with abnormal muscle tone. For example, Stinson et al.[8] recruited healthy participants to evaluate the effects of repositioning and sitting on the consistency of interface pressure. MacDonald et al. [9] and Kirby et al. [10] used able-bodied participants to compare the mean pressure and the wheelchair handling skills of a tilt-in-space wheelchair and a manual wheelchair equipped with a new rear anti-tip device from the perspectives of the caregiver and the user. Aissaoui et al. [11] used able-bodied subjects to compare the kinematic effects of conventional and compensatory legrests. However, these results still need to be verified for disabled subjects before they can be adopted into clinical practice. As reported above, when comparing the differences in back displacement caused by conventional and V-shaped seats at reclining positions, the BS of able-bodied subjects was found to differ from that of stroke patients with flaccid hemiplegia. Although many researchers study seating in wheelchairs for disabled people [12-14], there is very little research focused on stroke patients with flaccid hemiplegia. This paper is the first reported study that investigates the effect of V-shaped seats on the positioning of stroke patients with hypotonicity. In comparison to the 4-bar linkage [15], which was also designed to prevent patients from sliding in reclining wheelchairs and which proved to be effective in reducing back displacement for quadriplegic subjects, the V-shaped seat is a simpler yet effective design. Reclining wheelchairs with V-shaped seats are foldable, light-weight, easy to push, and inexpensive.

This study also demonstrates the trends of incremental forward sliding and increasing sacral sitting pressure when stroke patients repeatedly recline in both wheelchair types, indicating the importance of repositioning after returning the patients to an upright position even while using a wheelchair with a V-shaped seat. Moreover, we found that the overall peak pressure was often located on either the left or right side of subjects with flaccid hemiplegia, who were prone to sit with pelvic obliquity. The V-shaped seat mechanism alone could not lessen the high ischial pressure on patients who tended to sit on one side, possibly contributing to the insignificant difference in the mean pressures measured in the two types of wheelchair. Therefore, when seating a subject with flaccid hemiplegia, the pressure on the side to which the subject leans should be taken into account.

## Conclusions

In conclusion, V-shaped seats in reclining wheelchairs can help reduce forward sliding and the peak sacral pressure of stroke patients with flaccid hemiplegia, who are subject to more forward sliding and sacral pressure in reclining wheelchairs than able-bodied elders. V-shaped seats in reclining wheelchairs are a simple and effective design to help prevent the user from experiencing sacral sitting. The back displacement of able-bodied subjects when using both conventional and V-shaped seats in reclining positions differs from that of stroke patients with flaccid hemiplegia when using such seats. This paper is the first study to investigate the effect of V-shaped seats on the positioning of hemiplegic patients with hypotonicity. The results are of paramount value and should be considered when prescribing the use of reclining wheelchairs to subjects with flaccid hemiplegia.

## Competing interests

The authors declare that they have no competing interests. We declare that no party having a direct interest in the results of the research supporting this article has or will confer a benefit on us or on any organization with which we are associated.

## Authors' contributions

HCH conceived of the study, participated in its design and coordination, and drafted the manuscript. CHY participated in the design of the study and performed the statistical analysis. CMC and YSL were the main contributors to the data acquisition. KCC participated in the interpretation of the data and helped to draft the manuscript. All authors read and approved the final manuscript.

## Authors' information

HCH is the chief of the department of physical medicine and rehabilitation at Chia-Yi Christian Hospital and is also a Ph.D. student at the institute of biomedical engineering at National Cheng Kung University. CHY is the research assistant for HCH. CMC is the physical therapist at the assistive devices/technology center of the Chia-Yi Christian Hospital. YSL is the occupational therapist at the assistive devices/technology center of the Chia-Yi Christian Hospital. KCC is the director of the rehabilitation engineering laboratory at the institute of biomedical engineering at the National Cheng Kung University.
